# More efficient adaptation of cardiovascular response to repeated restraint in spontaneously hypertensive rats: the role of autonomic nervous system

**DOI:** 10.1038/s41440-024-01765-w

**Published:** 2024-07-01

**Authors:** Anna Vavřínová, Michal Behuliak, Martin Vodička, Michal Bencze, Peter Ergang, Ivana Vaněčková, Josef Zicha

**Affiliations:** Institute of Physiology, Czech Academy of Sciences, Prague, Czechia

**Keywords:** Adaptation, Adrenal glands, Habituation, Hypertension, Restraint stress

## Abstract

We hypothesized that sympathetic hyperactivity and parasympathetic insuficiency in spontaneously hypertensive rats (SHR) underlie their exaggerated cardiovascular response to acute stress and impaired adaptation to repeated restraint stress exposure compared to Wistar-Kyoto rats (WKY). Cardiovascular responses to single (120 min) or repeated (daily 120 min for 1 week) restraint were measured by radiotelemetry and autonomic balance was evaluated by power spectral analysis of systolic blood pressure variability (SBPV) and heart rate variability (HRV). Baroreflex sensitivity (BRS) was measured by the pharmacological Oxford technique. Stress-induced pressor response and vascular sympathetic activity (low-frequency component of SBPV) were enhanced in SHR subjected to single restraint compared to WKY, whereas stress-induced tachycardia was similar in both strains. SHR exhibited attenuated cardiac parasympathetic activity (high-frequency component of HRV) and blunted BRS compared to WKY. Repeated restraint did not affect the stress-induced increase in blood pressure. However, cardiovascular response during the post-stress recovery period of the 7th restraint was reduced in both strains. The repeatedly restrained SHR showed lower basal heart rate during the dark (active) phase and slightly decreased basal blood pressure during the light phase compared to stress-naive SHR. SHR subjected to repeated restraint also exhibited attenuated stress-induced tachycardia, augmented cardiac parasympathetic activity, attenuated vascular sympathetic activity and improved BRS during the last seventh restraint compared to single-stressed SHR. Thus, SHR exhibited enhanced cardiovascular and sympathetic responsiveness to novel stressor exposure (single restraint) compared to WKY. Unexpectedly, the adaptation of cardiovascular and autonomic responses to repeated restraint was more effective in SHR.

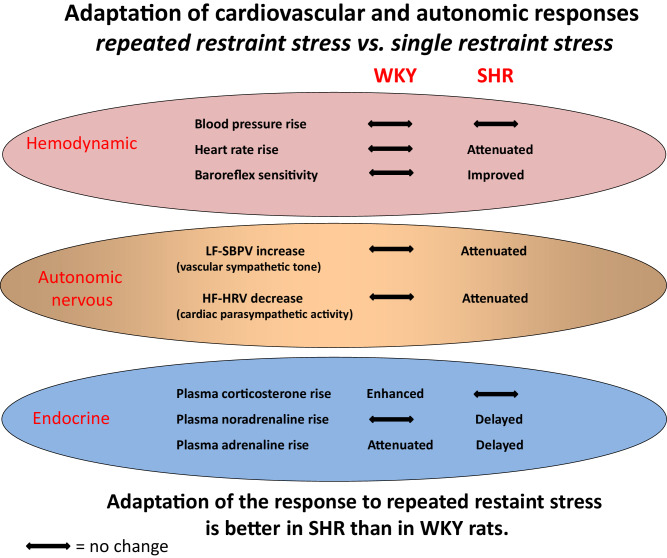

## Introduction

Physiological responses to stressor exposure are mediated primarily by the autonomic nervous system and hypothalamic-pituitary-adrenocortical (HPA) axis. The regulation of autonomic nervous system and HPA axis is complex and their appropriate interplay triggers a physiological response, which is adequate to the nature and intensity of the stressful stimulus [1]. The principal signaling molecules during stressful conditions are catecholamines (noradrenaline and adrenaline) released by the sympathetic nerve endings and adrenal medulla as well as glucocorticoids produced by adrenal cortex. Although the stress-induced release of glucocorticoids and catecholamines exerts temporary beneficial effects, their actions can be counterproductive or even deleterious under the conditions of prolonged or repeated stress. The progressive attenuation of the magnitude of cardiovascular, endocrine and behavioral responses elicited by repeated exposures to the same (homotypic) stressor is called adaptation or habituation. This important mechanism limits the maladaptive consequences of chronic stress and decreases the risk of pathophysiological changes by damping the responses to aversive stimuli that are not life-threatening [2, 3].

It is widely accepted that the stress-induced cardiovascular responses, characterized by increased blood pressure and heart rate, are predominantly triggered by the activation of the sympathetic nervous system [1, 4]. Conversely, the role of parasympathetic nervous system in stress-induced cardiovascular response is less studied. The interaction of sympathetic and parasympathetic branches of autonomic nervous system was “classically” described as reciprocal and antagonistic as seen during the baroreflex control of the heart rate, i.e., the decrease in blood pressure causes a parallel sympathetic activation and parasympathetic attenuation leading to tachycardia [5]. However, the revised concept of autonomic control described the coactivation of sympathetic and parasympathetic pathways under various conditions including stressor exposure [6]. Indeed, the activation of parasympathetic nervous system was demonstrated during the cardiovascular stress response elicited by restraint [7], auditory stimulus [8], or air puff stimulus [9]. The pattern of autonomic activation and resulting changes of heart rate and blood pressure depend on the novelty of the stimulus as well as on the rat strain used [10, 11]. This is also true for endocrine and behavioral responses to stress stimuli [12, 13]. The coactivation of both arms of autonomic nervous system allows more precise control of heart rate under the stress conditions [5, 6]. Makino et al. [14]. demonstrated that both arms of autonomic nervous system are also involved in circadian regulation of cardiovascular system. In normotensive Wistar rats, sympathectomy decreases blood pressure and heart rate during the dark active period, whereas the blockade of parasympathetic system by atropine treatment increased both abovementioned parameters especially during the light inactive period. Moreover, there is an increasing evidence in men as well as in rats that parasympathetic activation participates in the adaptation to psychological stressors and that the impaired parasympathetic activation might be related to pathophysiology of cardiovascular diseases [15, 16].

Spontaneously hypertensive rats (SHR), a widely used model of essential hypertension, are characterized not only by the augmented activity of sympathetic nervous system [17, 18] but also by the parasympathetic insuficiency [9, 19, 20]. Several papers [21, 22] indicated that adult SHR have higher values of blood pressure and heart rate in the dark period compared to the light period, while another study [23] did not not confirm this finding. Cardiovascular responses elicited by a single exposure to various stressful stimuli are augmented and prolonged in SHR compared to normotensive Wistar-Kyoto (WKY) rats [24, 25]. Besides an apparent stress hyperreactivity of SHR, SHR are able to adapt certain aspects of cardiovascular response to repeated exposure to various stressors, such as a graded reduction in the duration of restraint-induced tachycardia during repeated restraint [25, 26]. However, the pattern of activation of sympathetic and parasympathetic arms of autonomic nervous system during the single exposure to stressor or the adaptation to repeated stress are still unknown. In addition, it was proposed that the abnormal HPA function might contribute to the pathogenesis of hypertension in this strain because hypophysectomy as well as adrenalectomy performed in prehypertensive SHR prevented the development of high blood pressure [27]. Moreover, basal and stress-induced plasma corticosterone levels were shown to be higher in SHR than in WKY rats [4, 28]. Our previous study in normotensive Wistar rats showed an important permissive and/or stimulating role of glucocorticoids in the maintenance of sympathetic vasoconstriction and especially in the adequate response of cardiovascular system to stress exposure [29]. Glucocorticoids might influence cardiovascular tone by centrally mediated mechanisms, as the administration of corticosterone via implants above the dorsal hindbrain increased the basal blood pressure, reduced the gain of the heart rate baroreflex function and augmented the blood pressure response to restraint stress [30, 31]. In humans, a chronic exposure to glucocorticoids also increased the risk of cardiovascular alterations including hypertension [32, 33].

We hypothesized that SHR subjected to single restraint would exhibit exaggerated cardiovascular and hormonal responses (glucocorticoids and catecholamines) associated with the enhanced activation of sympathetic nervous system as well as with the reduced activity of parasympathetic nervous system as compared with normotensive WKY rats. In addition, we expected that the repeated exposure to restraint (chronic homotypic stress protocol) did not equally alter the pattern of autonomic responses in SHR and WKY rats thus contributing to the different adaptation of stress-induced cardiovascular response in both strains. The disclosure of strain-specific adaptation to chronic homotypic stress might change the point of view when interpreting cardiovascular experiments, especially in long-term studies comprising repeated measurements of blood pressure or other stressfull manipulation with animals.

## Methods

### Animals

Male Wistar-Kyoto rats (WKY) and spontaneously hypertensive rats (SHR) aged 18–24 weeks were obtained from the breeding colony of the Institute of Physiology, Czech Academy of Sciences (CAS), Prague. Rats were housed in a temperature-controlled room (23 ± 1 °C), with a 12/12 h light/dark cycle, with *ad libitum* access to water and standard rat chow (Altromin 1324, Altromin, Germany). All procedures and experimental protocols were approved by the Ethical Committee of the Institute of Physiology CAS, and were conducted in accordance with European Convention for the Protection of Vertebrate Animals used for Experimental and other Scientific Purposes (Protocol Nr. 72/2016).

### Restraint stress

Restraint was performed between 10 a.m. and 12:00 p.m. (noon). The rats were placed into the transparent plastic cylinders (6.5 cm inner diameter; adjustable in length depending on the animal size) equipped with ventilation holes. The rats in the single stress protocol were subjected to 120 min restraint once, whereas the rats in the repeated stress protocol were exposed to daily 120 min restraint for seven consecutive days [11]. Stress-naive rats, which were not subjected to any restraint, were used as control group in single stress protocol. On the other hand, the rats subjected to 120 min restraint for 6 consecutive days and to subsequent 1 day rest were used as adapted controls in the repeated stress protocol. The term “acute stress” refers to the immediate response to the 1st or 7th exposure to restraint in single or repeated stress protocol, respectively. The values determined during the last 7th restraint were considered to represent the chronic releated stress.

### Radiotelemetry

WKY and SHR were implanted with telemetry devices (model HD-S10, Data Sciences International, USA) under isoflurane anesthesia (5% for induction and 2.5% for maintenance; Forane, AbbVie, USA) as previously described [29, 34, 35]. Briefly, a midline abdominal incision was made and the tip of the telemetry probe catheter was inserted retrogradely into the abdominal aorta through a puncture site which was subsequently sealed with tissue adhesive (Vetbond™, 3M Animal Care Products, USA). The body of transmitter was secured to the abdominal wall before closing the midline incision by Michel suture clips (Medin, Czech Republic). Transmitted data were recorded and analyzed using the Dataquest A.R.T. System (Data Sciences International, USA). Following a 10-day recovery period, blood pressure (BP), heart rate (HR), body temperature and locomotor activity were measured in freely moving rats for 3 days (5-min intervals were recorded every 15 min four times per hour) and average values during the light inactive phase and the dark active phase were computed. Thereafter, the rats were exposed to daily 120 min restraint for seven consecutive days. For a better time-resolution of stress-induced changes, the recording protocol was changed (2-min intervals recorded every 5 min 12 times per hour). After the last stress session, the telemetric measurement in freely moving animals continued for the next 3 days.

### Power spectral analysis of systolic blood pressure variability and heart rate variability

Power spectral analysis of systolic blood pressure variability (SBPV) and heart rate variability (HRV) was calculated as previously described [34]. Systolic blood pressures (SBP) were derived on a beat-to-beat basis by The Blood Pressure Add-On for LabChart software (ADInstruments Ltd, Australia) from the original direct BP recordings. Time-domain indices of HRV, the standard deviation of normal inter-beat intervals (SDNN), and the root mean square of successive inter-beat interval differences (RMSSD) were estimated. RMSSD was used as the primary time-domain measure to estimate the vagally mediated changes reflected in HRV [36]. Beat-to-beat series of SBP and inter-beat interval data were used for frequency-domain measurements of SBPV and HRV based on the estimation of power spectral density by the fast Fourier transform-based Welch’s method and Burg autoregressive method. Very low-frequency (VLF; 0.02–0.2 Hz), low-frequency (LF; 0.2–0.75 Hz), and high-frequency (HF, 0.75–4 Hz) components of SBPV and HRV were determined. LF-SBPV represents a marker of vascular sympathetic activity, whereas HF-HRV represents a marker of cardiac parasympathetic activity [37, 38].

### Baroreceptor-heart rate reflex and heart rate response to methylatropine

Baroreceptor-HR reflex sensitivity in rats subjected to single or repeated stress protocol was measured by a modified pharmacological Oxford technique as described previously [34]. One day before BP measurement, polyethylene catheters were implanted into the left carotid artery (PE50) and jugular vein (PE10) under isoflurane anesthesia. Both catheters were filled with heparinized saline, tunneled subcutaneously and exteriorized in the interscapular region. BP was measured using the PowerLab System (ADInstruments, Australia) in conscious restrained rats during the 1^st^ or 7^th^ restraint session. Vasodepressor NO donor (sodium nitroprusside) and vasopressor α_1_-adrenergic agonist (phenylephrine) were administered in increasing doses (2.5–20 µg kg^−1^ and 1.25–10 µg kg^−1^, respectively) to alter BP over a wide range and to produce reflex tachycardia or bradycardia, respectively. Baroreflex sensitivity was calculated as the slopes of regression lines from either nitroprusside-induced BP reduction accompanied by a reflex tachycardia or from phenylephrine-induced BP elevation associated with a reflex bradycardia. At the end of the stress session, cardiac vagal tone was also measured as a magnitude of tachycardic response induced by methylatropine administration (2 mg kg^−1^) [39]. Baroreflex sensitivity was also determined from telemetric recordings by spontaneous sequences technique [40] using Hemolab software ver. 21.0 as previously described [35, 41].

### Blood sampling and determination of plasma corticosterone, aldosterone, and catecholamines levels

Plasma samples were collected from the rats subjected to single or repeated stress protocol. Separate groups of rats were used for particular time-points (*n* = 8 for each strain): controls, 10 min of restraint or 120 min of restraint. The rats were rapidly anesthetized with isoflurane which does not influence basal and stress-induced corticosterone levels [42]. Blood was collected from abdominal aorta into EDTA containing tubes (S-Monovette® K3E tubes, Sarstedt, Germany) and centrifuged for 10 min (3000 *g*, 4 °C). The animals were sacrificed by exsanguination, adrenal glands and thymus were weighed. Plasma corticosterone and aldosterone levels were measured using Corticosterone rat/mouse ELISA (LDN, Germany) and Aldosterone ELISA (LDN), respectively. Plasma concentrations of catecholamines (noradrenaline and adrenaline) and their metabolites (normetanephrine and metanephrine) were measured using 2-CAT (N-D) Research ELISA and 2-MET Plasma ELISA Fast Track (LDN).

### Statistical analysis

The data are expressed as the means ± SEM. Multiple-group comparisons were performed by two-way ANOVA with the main effects of *stress chronicity* (single vs. repeated stress protocol) and *strain* (WKY vs. SHR). For the comparison of time courses of telemetrically measured BP, HR, body temperature, SBPV and HRV, we used two-way repeated measures ANOVA with the main effect of *stress protocol* (single vs. repeated stress protocol) and *time* (within-animal repeated factor), separately for WKY and SHR. Plasma hormone levels were analyzed in WKY and SHR using two-way ANOVA with the main effects of *stress chronicity* (single vs. repeated stress protocol) and *acute stress exposure* (control vs. 10 min restraint or 120 min restraint). Follow-up comparisons of the means comprising the main effects or their interaction were conducted using Bonferroni *post-hoc* test. The differences were considered to be significant at *p* < 0.05.

## Results

### Body weight and organ weights in stress-naive (unstressed) and repeatedly stressed rats

SHR exhibited lower body weight (b.w.) than WKY rats irrespectively of chronic stress exposure (stress-naive WKY 329 ± 4 and SHR 315 ± 3 g, chronically stressed WKY rats 323 ± 7 and SHR 312 ± 4 g; stress chronicity: NS, strain: *p* < 0.05, interaction: NS). The relative weight of adrenal glands was greater in SHR and it was increased by the chronic stress exposure in both strains (stress-naive WKY 10.9 ± 0.3 and SHR 13.0 ± 0.2 mg/100 g b.w., chronically stressed WKY rats 13.8 ± 0.2 and SHR 15.0 ± 0.4 mg/100 g b.w.; stress chronicity: p < 0.01, strain: *p* < 0.01, interaction: NS). The increase in the relative adrenal weight was less pronounced in SHR than in WKY (WKY + 27 ± 1%, SHR + 15 ± 3%; *p* < 0.01). The relative thymus weight was lower in SHR and it was decreased by the chronic stress exposure in both strains (stress-naive WKY 99.4 ± 2.1 and SHR 75.4 ± 3.2 mg/100 g b.w., chronically stressed WKY rats 74.5 ± 2.9 and SHR 60.8 ± 4.1 mg/100 g b.w.; stress chronicity: *p* < 0.01, strain: *p* < 0.01, interaction: NS). Repeatedly restrained rats of both strains exhibited typical markers of chronic stress - adrenal hypertrophy and thymic involution. The increase in relative adrenal weight was more pronounced in WKY rats.

### Basal cardiovascular and autonomic parameters in stress-naive and repeatedly stressed rats

**Fig. 1:**
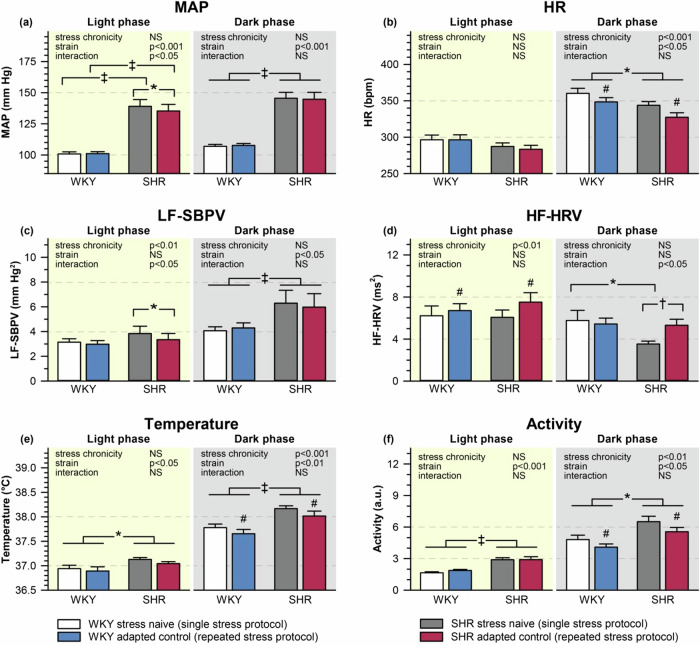
Basal mean arterial pressure (MAP), heart rate (HR), autonomic parameters, core body temperature, and locomotor activity in freely moving stress-naive and chronically stressed Wistar-Kyoto (WKY) and spontaneously hypertensive rats (SHR). MAP (**a**), HR (**b**), the low-frequency component of systolic blood pressure variability (LF-SBPV) (**c**), the high-frequency component of heart rate variability (HF-HRV) (**d**), body temperature (**e**) and locomotor activity (**f**) are shown as means ± SEM during the light and the dark phase of the day; *n* = 8 for each group. *P < 0.05; †*P* < 0.01; ‡*P* < 0.001 vs particular group; #*P* < 0.05 for the main effect of stress chronicity; NS non-significant

Blood pressure and heart rate were measured in freely-moving rats by radiotelemetry, whereas the activity of autonomic nervous system was estimated by the analysis of systolic blood pressure variability and heart rate variability. Basal mean arterial pressure (MAP), locomotor activity and core body temperature were higher in stress-naive SHR compared with WKY rats (Fig. 1a, e, f). There was a moderate MAP elevation and a pronounced HR increase in the dark period compared to the light period (WKY: 107 ± 2 vs. 101 ± 2 mmHg; 361 ± 6 vs. 294 ± 7 bpm; SHR: 147 ± 3 vs. 140 ± 2 mmHg; 345 ± 6 vs. 288 ± 5 bpm). During the dark phase, higher blood pressure in SHR is associated with higher low-frequency component of SBPV (LF-SBPV; a marker of vascular sympathetic activity; Fig. 1c). Repeated stress did not affect basal MAP in WKY rats, whereas repeatedly stressed SHR exhibited slightly lower MAP during the light phase, which corresponds to decreased LF-SBPV compared to stress-naive SHR (Fig. 1a, c). The basal high-frequency component of HRV (HF-HRV; a marker of cardiac parasympathetic activity) was lower in stress-naive SHR compared with WKY rats during the dark phase (Fig. 1b, d). The reduction of basal HR in repeatedly stressed SHR during the dark phase corresponds to an increase in HF-HRV (Fig. 1b, d) and also to an increase in RMSSD (the root mean square of successive inter-beat interval differences; the primary time-domain measure used to estimate the vagally mediated changes reflected in HRV). In both strains repeated stress increased HRV indicating enhanced parasympathetic tone during chronic stress exposure (Supplementary Table S1).

Thus, repeated restraint lowered basal heart rate due to increased parasympathetic tone in freely-moving rats of both strains. Furthermore, repeatedly restrained SHR showed moderately reduced vascular sympathetic tone accompanied by decreased blood pressure during the light period.

### Changes of cardiovascular and autonomic parameters during single or repeated restraint

**Fig. 2:**
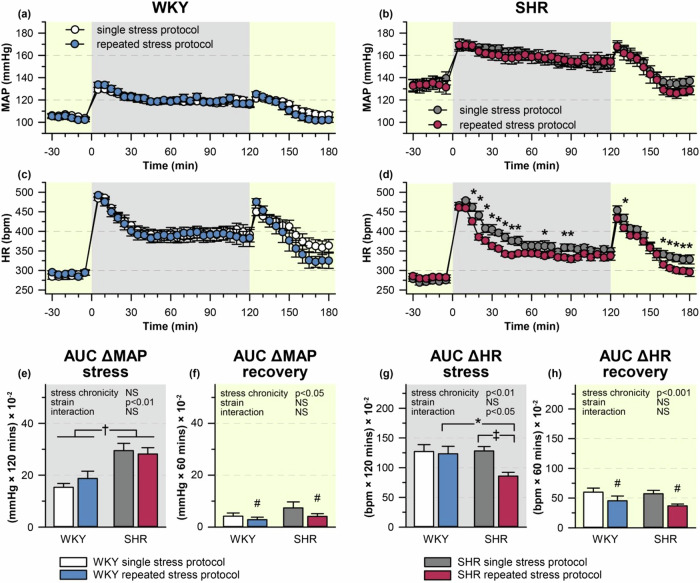
Mean arterial pressure (MAP) and heart rate (HR) during restraint. WKY and SHR were subjected to 120 min restraint (marked in gray) either once (single stress protocol) or for seven consecutive days (repeated stress protocol). The time course of changes in MAP (**a**, **b**), HR (**c**, **d**) were measured by radiotelemetry and averaged over 5-min intervals; each point represents a group mean ± SEM; *n* = 8 for each group. Area under the curve (AUC) of ΔMAP (**e**, **f**) and ΔHR (**g**, **h**) during the stress session (**e**, **g**) and post-stress recovery period (**f**, **h**). *P < 0.05; †*P* < 0.01; ‡*P* < 0.001 vs particular group; NS non-significant

**Fig. 3:**
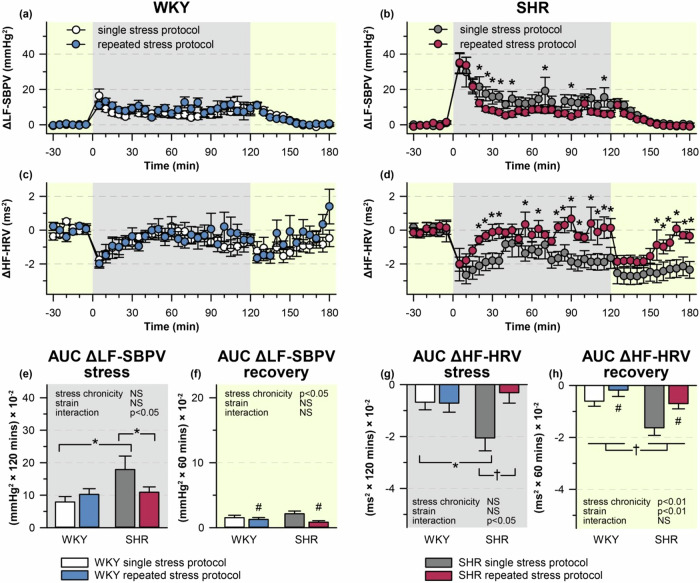
Low-frequency component of systolic blood pressure variability (LF-SBPV) and high-frequency component of heart rate variability (HF-HRV) during restraint. WKY and SHR were subjected to 120 min restraint (marked in gray) either once (single stress protocol) or for seven consecutive days (repeated stress protocol). The time course of changes in LF-SBPV (**a**, **b**) and HF-HRV (**c**, **d**) were computed by power spectral analysis; each point represents a group mean ± SEM; *n* = 8 for each group. Area under the curve (AUC) of ΔLF-SBPV (**e**, **f**) and ΔHF-HRV (**g**, **h**) during the stress session (**e**, **g**) and post-stress recovery period (**f**, **h**). *P < 0.05; †*P* < 0.01; ‡*P* < 0.001 vs particular group; NS non-significant

During the acute restraint session, SHR subjected to a single restraint showed an augmented increase in MAP (Fig. 2a, b, e) and LF-SBPV (Fig. 3a, b, e) compared with WKY rats. Repeated stress exposure did not affect stress-induced MAP response in either strain during the 7th stress session (Fig. 2e). However, MAP response during the post-stress recovery period of the 7th restraint session was reduced in rats of both strains when compared with the single stress protocol (Fig. 2f). This adaptation to repeated stress was accompanied by a decrease in LF-SBPV during the post-stress recovery period (Fig. 3f). Moreover, repeated stress also led to the attenuation of stress-induced LF-SBPV response during the 7th restraint session in SHR (Fig. 3b, e).

HR increase induced by a single restraint was not significantly different between SHR and WKY rats throughout the stress session or post-stress recovery period (Fig. 2c, d, g, h). In WKY rats, HF-HRV (a marker of cardiac parasympathetic activity) was initially decreased by the exposure to acute restraint but it returned towards the baseline values (Fig. 3c). SHR subjected to a single restraint exhibited reduction of HF-HRV which did not return to the baseline level during the stress session or the recovery period (Fig. 3d, g, h). Repeatedly restrained SHR showed a significant decrease in stress-induced tachycardia (Fig. 2d, g) which is in line with the faster return of HF-HRV to baseline values (Fig. 3d, g).

The acute stress-induced increase in body temperature was more pronounced in SHR than in WKY rats during both single and repeated stress protocols (Supplementary Fig. 1). Repeated stress exposure did not affect stress-induced body temperature response in either strain during the 7^th^ stress session. Attenuated MAP and HR responses during the post-stress period of chronically stressed animals were not accompanied by a decrease in locomotor activity (Supplementary Fig. 1).

Taken together, SHR subjected to a single restraint showed augmented vascular sympathetic activity and a more pronounced attenuation of parasympathetic activity compared to WKY rats. Repeated restraint weakened stress-induced autonomic response in SHR, thereby abolishing the inter-strain difference during the 7th session of restraint. However, both SHR and WKY rats exhibited the adaptation of autonomic and cardiovascular responses during the post-stress recovery period.

### Plasma levels of stress hormones

**Fig. 4:**
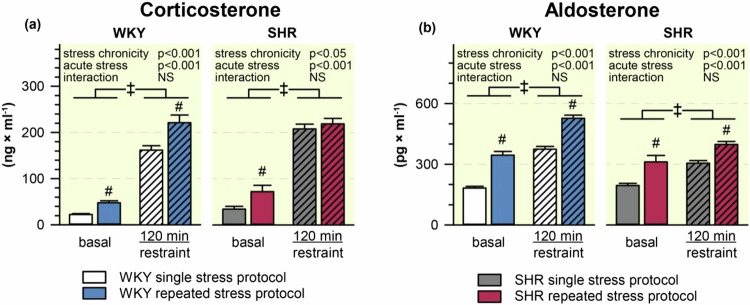
Basal and stress-induced plasma levels of corticosterone (**a**) and aldosterone (**b**) in WKY and SHR subjected to single or repeated stress protocol. The data are shown as a mean ± SEM; *n* = 8 for each group. *P < 0.05; †*P* < 0.01; ‡*P* < 0.001 vs particular group; #*P* < 0.05 for the main effect of stress chronicity; NS non-significant

The basal corticosterone levels were higher in stress-naive SHR compared with WKY controls and the repeated restraint elevated basal corticosterone levels in animals of both strains (stress-naive: WKY 22.3 ± 2.2 and SHR 34.5 ± 5.9 ng/ml, chronically stressed: WKY 47.8 ± 4.4 and SHR 72.3 ± 13.6 ng/ml; stress chronicity: *p* < 0.001, strain: *p* < 0.05, interaction: NS). The acute restraint increased plasma corticosterone in both strains during single and repeated stress protocol. Stress-induced plasma levels of corticosterone were greater in SHR compared to WKY during the single restraint protocol. Unexpectedly, the repeated stress augmented an acute increase of plasma corticosterone in WKY rats but not in SHR (Fig. 4a). The basal aldosterone levels were similar in stress-naive SHR and WKY rats and the repeated stress elevated them in both strains (stress-naive: WKY 182.5 ± 8.7 and SHR 195.4 ± 10.3 pg/ml, chronically stressed: WKY rats 344.7 ± 18.6 and SHR 312.2 ± 31.4 pg/ml; stress chronicity: *p* < 0.001, strain: NS, interaction: NS). The acute restraint increased plasma aldosterone in both single and repeated stress protocols, the effect being smaller in SHR (Fig. 4b).

**Fig. 5:**
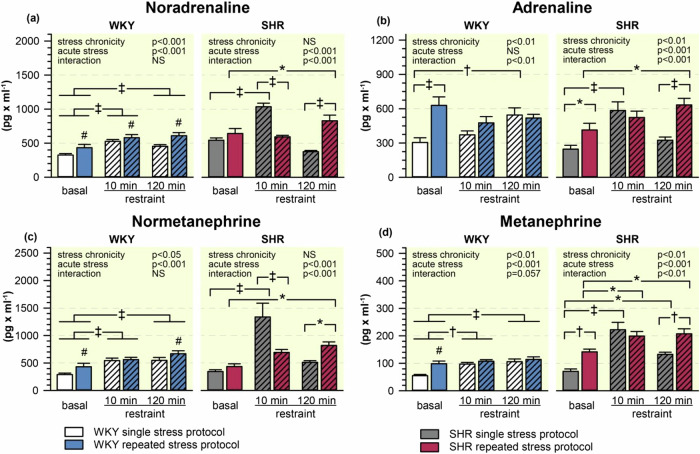
Basal and stress-induced plasma levels of noradrenaline (**a**), adrenaline (**b**), normetanephrine (**c**), and metanephrine (**d**) in WKY and SHR subjected to single or repeated stress protocol. The data are shown as a mean ± SEM; *n* = 8 for each group. *P < 0.05; †*P* < 0.01; ‡*P* < 0.001 vs particular group; #*P* < 0.05 for the main effect of stress chronicity; NS non-significant

The basal plasma levels of noradrenaline were higher in stress-naive SHR compared to WKY rats and the repeated stress elevated them in both strains (stress-naive: WKY 325.6 ± 22.6 and SHR 546.1 ± 33.4 pg/ml, chronically stressed: WKY rats 434.4 ± 48.0 and SHR 644.0 ± 72.8 pg/ml; stress chronicity: *p* < 0.05, strain: *p* < 0.001, interaction: NS). Plasma noradrenaline levels were elevated more in SHR than in WKY rats after 10 min of acute restraint but they were similar at the end of restraint session. This early peak of plasma noradrenaline was not observed in the repeatedly stressed SHR in which elevated plasma noradrenaline level was found after 120 min of stress (Fig. 5a). The above described findings were largely confirmed by similar changes in plasma normetanephrine levels (Fig. 5c). The basal plasma levels of adrenaline (a biochemical marker of adrenal medulla activity) were lower in SHR compared to WKY rats while repeated stress elevated basal plasma adrenaline in both rat strains (stress-naive: WKY 305.3 ± 40.9 and SHR 246.5 ± 33.9 pg/ml, chronically stressed: WKY rats 629.7 ± 73.8 and SHR 414.7 ± 58.1 pg/ml; stress chronicity: *p* < 0.001, strain: *p* < 0.05, interaction: NS). During the acute restraint, there was a gradual increase of plasma adrenaline levels in WKY rats subjected to a single stress protocol but not in the repeatedly stressed WKY rats. In contrast, the acute restraint elicited an early plasma adrenaline increase only in SHR subjected to a single stress protocol, whereas there was a late plasma adrenaline increase in the repeatedly stressed SHR (Fig. 5b). Basal plasma metanephrine levels were elevated in the repeatedly stressed animals of both strains. The pattern of changes in plasma metanephrine levels corresponds to the observed plasma adrenaline changes (Fig. 5d).

Thus, SHR showed a more pronounced rise in plasma corticosterone and noradrenaline during single restraint than WKY rats. Stress-induced rise in plasma adrenaline was faster in SHR, but the magnitude of this increase was similar in both strains. Repeated restraint elevated basal plasma levels of all measured stress-related hormones in SHR and WKY rats. The adaptation of acute endocrine response to chronic stress was strain-specific.— Plasma corticosterone rise was increased in WKY rats but unchanged in SHR, plasma noradrenaline rise was similar in WKY rats but delayed in SHR, and plasma adrenaline rise was diminished in WKY rats but delayed in SHR.

### Baroreceptor-heart rate reflex and heart rate response to methylatropine

**Fig. 6:**
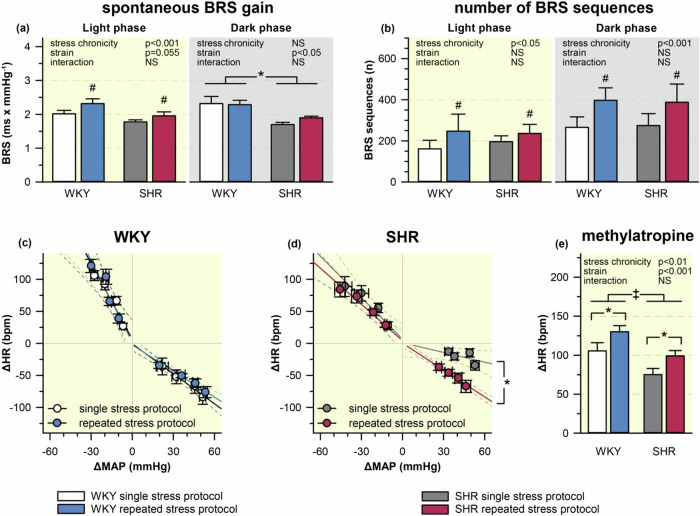
Baroreflex sensitivity (BRS) and heart rate (HR) response to methylatropine in WKY and SHR subjected to single or repeated stress protocol. Baroreceptor-heart rate reflex sensitivity was measured either by spontaneous sequence technique (**a**, **b**) or by modified pharmacological Oxford technique (**c**, **d**). The slopes of regression lines were calculated separately for the nitroprusside-induced tachycardia and for the phenylephrine-induced bradycardia. The HR response to the administration of muscarinic acetylcholine receptors antagonist methylatropine indicates cardiac vagal tone (**e**). The data are shown as means ± SEM; *n* = 8 for each group. *P < 0.05; †*P* < 0.01; ‡*P* < 0.001 vs particular group; NS non-significant

Baroreceptor-HR reflex sensitivity (BRS) measured by spontaneous sequence technique in the dark phase was lower in SHR compared with WKY rats (Fig. 6a). The single restraint significantly reduced spontaneous baroreflex sensitivity during the stress session in both strains (WKY 1.4 ± 0.2 ms/mmHg, SHR 1.2 ± 0.1 ms/mmHg), whereas the repeated stress partially restored the spontaneous baroreflex sensitivity measured during the restraint session in both strains (WKY 1.7 ± 0.1 ms/mmHg, SHR 1.6 ± 0.1 ms/mmHg). The number of spontaneous baroreflex events was similar in the stress-naive SHR and WKY rats and chronic stress increased this parameter in both strains (Fig. 6b).

The baroreceptor-HR reflex sensitivity in rats subjected to single or repeated stress protocol was also measured by modified pharmacological Oxford technique. The slope of tachycardic response to sodium nitroprusside was less steep in SHR than in WKY rats irrespective of the stress protocol (Fig. 6c, d; single stress protocol WKY −4.01 ± 0.29 and SHR −2.51 ± 0.53 bpm/mmHg, repeated stress protocol WKY −4.02 ± 0.36 and SHR −1.90 ± 0.38 bpm/mmHg; stress chronicity: NS, strain: *P* < 0.001, interaction: NS). The slope of bradycardic response to phenylephrine was less steep in SHR subjected to a single restraint in comparison with WKY rats (Fig. 6c, d; single stress protocol WKY −1.62 ± 0.32 and SHR −0.59 ± 0.17 bpm/mmHg). The repeatedly stressed SHR (but not WKY rats) exhibited steeper slope of bradycardic response to phenylephrine (vagal component of baroreceptor-HR reflex) compared to single stress protocol (repeated stress protocol WKY −1.38 ± 0.19 and SHR −1.41 ± 0.27 bpm/mmHg; stress chronicity: NS, strain: *P* = 0.06, interaction: *P* < 0.05).

Cardiac vagal tone, which was measured as a magnitude of tachycardic response induced by methylatropine administration, was smaller in SHR than in WKY rats. The repeated stress augmented HR response to methylatropine in both strains (Fig. 6e), indicating the enhancement of parasympathetic tone by repeated stress.

Spontaneous sequence technique (evaluating baroreflex on the basis of physiological changes of blood pressure and heart rate) revealed that repeated stress improved baroreflex in both rat strains. In contrast, pharmacological Oxford method (measuring cardiovascular changes elicited by vasopressor or vasodilator drugs) indicated the improvement of baroreflex sensitivity only in repeatedly restrained SHR, in which bradycardic response to phenylephrine was enhanced due to the potentiation of vagal arm of baroreflex. The potentiation of cardiac vagal tone in repeatedly stressed rats of both strains was also indicated by the augmentation of tachycardic response to methylatropine.

## Discussion

The novelty of our study is an attempt to compare the cardiovascular and neuroendocrine adaptation of normotensive and hypertensive rats to repeated homotypic stress. We hypothesized that autonomic dysregulation in spontaneously hypertensive rats may underlie their exaggerated cardiovascular response to acute stress and impaired adaptation to repeated restraint compared to normotensive Wistar-Kyoto rats. SHR subjected to a single restraint exhibited augmented pressor response, similar tachycardia, enhanced vascular sympathetic tone, attenuated cardiac parasympathetic activity as well as higher stress-induced rise in plasma corticosterone and catecholamines as compared with acutely restrained WKY rats. Repeated restraint exposure did not affect stress-induced blood pressure increase, but reduced cardiovascular response during post-stress recovery period in both strains. The adaptation of SHR (but not WKY rats) to repeated restraint was characterized by attenuated stress-induced tachycardia, augmented cardiac parasympathetic activity, reduced vascular sympathetic activity, and improved baroreflex sensitivity compared to animals subjected to a single restraint only. Thus, SHR exhibited enhanced cardiovascular and sympathetic responsiveness to novel stressor exposure but their adaptation of cardiovascular and autonomic responses to chronic homotypic stress was more effective compared to WKY rats.

Stress-induced cardiovascular response is generally mediated by the autonomic nervous system. We observed augmented stress-induced increase in BP and LF-SBPV in SHR subjected to a single restraint compared with WKY rats, which is in line with the well-known sympathetic hyperactivity in SHR [17, 18]. The sympathetic nervous system is also involved in body temperature regulation through its effects on cutaneous vasoconstriction and heat production by brown adipose tissue [43, 44]. Indeed, we observed a more pronounced stress-induced hyperthermia in SHR than in WKY rats, which is in accordance with the previous report [45]. We have earlier demonstrated that stress-induced cardiovascular response was diminished in sympathectomized SHR and WKY rats [35] and their temperature response was also markedly reduced (Behuliak and Vavřínová, unpublished data). The stress-induced pressor responses were not changed by the repeated exposure to homotypic stressor in either SHR or WKY rats, which is in agreement with the study performed by McDougall et al. [26]. On the other hand, we observed the adaptation of cardiovascular responses (pressor response and tachycardia) during the post-stress recovery period of the 7th restraint session, which is in accordance with the previous study performed in normotensive Wistar rats [44]. We observed the attenuation of stress-induced rise of LF-SBPV as well as a less pronounced rise of plasma noradrenaline level in the repeatedly stressed SHR, which might be in contrast with their unchanged pressor response. However, the sympathetic vasoconstriction might be partially replaced by other mechanisms in these animals. Sympathoadrenal system activation seems to be a probable candidate for such mechanism since it mediates a sustained pressure response during the acute restraint session [7]. Indeed, we observed increased basal plasma adrenaline levels in the repeatedly restrained rats of both strains. There was also a significant increase in plasma adrenaline level at the end of the 7^th^ restraint session in the repeatedly stressed SHR. Moreover, our previous study in sympathectomized SHR and WKY rats [35] showed that a major enhancement of vascular sensitivity to adrenaline together with its increased plasma levels plays an important compensatory role in BP maintenance. Our results support the idea of Crestani [5] that during stress the total peripheral resistance is increased in hypertensive but not in normotensive rats. On the other hand, stress-induced elevation of cardiac output seems to be more important for BP increase in normotensive animals subjected to acute stress exposure.

The repeated restraint exposure elevated basal corticosterone levels, which is consistent with previous studies in chronically stressed animals [11, 46]. The repeated stress augmented an acute stress-induced increase of plasma corticosterone in WKY rats, whereas there was no change in SHR between the single and repeated stress protocol. Higher basal corticosterone levels observed in the repeatedly stressed rats of both strains might also contribute to the stress-induced pressor response because the sensitivity of the cardiovascular system to pressor effects of noradrenaline depends on permissive and/or stimulative role of glucocorticoid signaling [29, 47]. This is in contrast with the previously reported reduction of HPA axis activation during the repeated restraint in Sprague-Dawley, Wistar, Fischer 344 and Lewis rats [11, 46, 48].

Our analysis of heart rate variability (Supplementary Table 1) revealed similar HF-HRV in SHR and WKY rats during the light (inactive) phase of the day, whereas during the dark (active) phase there was a lower HF-HRV in SHR than in WKY rats. Thus, in SHR the dark active phase was associated with enhanced sympathetic activity, while parasympathetic activity was attenuated. This is not seen in WKY rats in which sympathovagal balance (LF-HRV/HF-HRV ratio) was almost unchanged during the day and night. The altered function of parasympathetic nervous system was reported in SHR [9, 19]. Although the initial decrease of cardiac parasympathetic activity at the onset of acute stress exposure was observed in all experimental groups, the subsequent activation of parasympathetic tone during the stress session and recovery period was considerably reduced in SHR subjected to single restraint. Cardiovascular recovery from stress is associated with increased vagal modulation despite residual sympathetic activation. Vagal rebound may be involved in mechanisms resetting the baroreflex sensitivity. Diminished vagal rebound during the recovery from stress is associated with standard risk factors for cardiovascular disease [16]. The attenuation of parasympathetic activity in SHR was also confirmed by decreased HR response to methylatropine (blockade of muscarinic acetylcholine receptors) in comparison with WKY rats. However, SHR and WKY rats subjected to single restraint exhibited similar tachycardia, which contrasts with a previous study describing augmented HR response in SHR compared to WKY rats [26]. The discrepancy between the altered autonomic response but unchanged HR response to acute stress might be ascribed to different adjustment of cardiac conduction system to sympathetic hyperactivation in SHR. Heart rate and cardiac output are increased in prehypertensive SHR but both parameters are normalized during the later ontogenesis [49, 50]. Indeed, it was demonstrated that isolated working heart or atria of adult SHR exhibited lower basal heart rate [51, 52] which might represent the compensation of higher sympathetic stimulation in vivo. In the present study, we demonstrated that the attenuation of stress-induced HR response in the repeatedly stressed SHR (but not in WKY rats) was accompanied by a potentiation of HF-HRV as compared to single-restrained SHR. This is in line with a previously reported reduction of stress-induced HR response in repeatedly immobilized SHR [4]. Moreover, repeated stress decreased basal HR during the dark phase of the day in both SHR and WKY rats. This effect persisted more than 3 days after the last restraint session and thus cannot be ascribed to a decreased locomotor activity (Behuliak, data not shown). A substantial decrease in HR enduring beyond the duration of the stress exposure was described earlier [53, 54]. So called “enduring vagal rebound”, referring to a prolonged increase in the resting vagal drive, has been interpreted as a sign of adaptation that overcomes the commonly observed stress-induced sympathetic hyperactivity. In the present study, the potentiation of cardiac vagal tone in the repeatedly stressed rats was demonstrated by the increase in heart rate response to the administration of muscarinic acetylcholine receptor antagonist methylatropine in both strains. Moreover, chronic stress improved baroreceptor-heart rate reflex efficiency during the light phase of the day in rats of both strains. Repeated stress also increased total HRV in both strains, which indicated overall changes in cardiac autonomic control during chronic stress exposure associated with increased baroreflex responsiveness, which was evident from a greater number of baroreflex events. Baroreflex function might also be affected by corticosterone levels as the number of baroreflex events was reduced after surgical or pharmacological adrenalectomy [29, 55]. Baroreceptor-heart rate reflex efficiency (either calculated from spontaneous or pharmacologically-induced BP changes) was blunted in SHR, which is in line with previous reports [34, 56]. The repeatedly stressed SHR showed the augmentation of baroreflex sensitivity (bradycardic response to the administration of phenylephrine). Our data indicate that vagal rebound is a common adaptive mechanism to chronic stress elicited by the repeated exposure to homotypic stressor. This parasympathetic activation caused a restoration of distinct cardiovascular abnormalities of stress response in SHR, e.g., baroreflex sensitivity or HR response to stress exposure.

The exaggerated responsivity to novel stressors is a common feature of chronically stressed individuals [3]. Adult stress-naive SHR demonstrated adrenal hypertrophy, thymic atrophy, hyperthermia, increased locomotor activity and higher plasma corticosterone levels. These abnormalities might be considered as a consequence of chronic exposure to various environmental stressors during the ontogenesis [1]. It was proposed that SHR are resilient to the anxiogenic effects of early life trauma (maternal separation) because they have a genetic predisposition to develop hyperactivity rather than anxiety and depression to a novel environment as compared to WKY rats [57]. Higher basal corticosterone levels and altered ACTH response to corticotropin-releasing hormone in SHR were described already at the age of 5–6 weeks [58]. Thus, the genetic predisposition to the abnormalities of central stress system in SHR might determine the stress vulnerability already in the early ontogenesis and can thus contribute to the development of enhanced autonomic and cardiovascular response to novel stressors in adult SHR. Our results suggest greater autonomic, humoral and cardiovascular adaptation to repeated exposure to homotypic stressor in SHR as compared to WKY rats, which is in line with a previous study done by Kvetnansky et al. [4]. In addition, a more pronounced enlargement of adrenals was observed in WKY rats indicating their less effective adaptation to chronic homotypic stress. Indeed, it was previously shown that not only SHR but also WKY rats can be considered as hyper-responsive to stress in comparison with other normotensive strains (e.g., Wistar or Sprague-Dawley rats) [59, 60], although their coping style is different - SHR displaying typical behavioral activation, whereas there is a freezing in WKY rats [61, 62].

Therefore, SHR are considered as a model of attention deficit/hyperactivity disorder (ADHD) [63–65], while WKY rats are characterized by anxiety- and depressive-like behavioral phenotype according to various behavioral tests e.g., forced swimming tests [62, 66–68]. Basal corticosterone levels of WKY rats do not differ from other strains [12, 67, 69]. Although there is no apparent relationship between behavior and stress-induced levels of corticosterone [70], WKY rats often display exaggerated stress-induced HPA axis response compared to other strains [12, 71], which is associated with slower recovery after an acute stress [61, 72, 73]. This is in accordance with our results showing less effective adaptation of the autonomic and cardiovascular response to repeated restraint stress in WKY rats compared to SHR rats. The delayed recovery often reflects the perceived severity of uncontrollable stress [74]. Moreover, rats usually respond to repeated homotypic stressor by decreasing the corticosterone response [48]. The lack of adaptation of corticosterone to repeated restraint in WKY rats further highlights the vulnerability of WKY strain to stressors.

The above mentioned strain differences in behavior may question the use of WKY rats as controls for studying the adaptation of cardiovascular response to stress. However, WKY rats are often used as a standard control inbred strain in the research of genetic hypertension. They differ considerably in many important hemodynamic, vascular, and autonomic nervous parameters from SHR. In the field of cardiovascular research, the role of stress reactivity of particular rat strain in the regulation of blood pressure and heart rate is often underestimated. Our present study was designed to evaluate cardiovascular, autonomic nervous, and endocrine adaptation to repeated restraint in normotensive and hypertensive animals. We demonstrated that strain-specific adaptation to chronic homotypic stress comprises autonomic, cardiovascular, and endocrine changes of basal values in freely moving rats as well as in the response of animals subjected acutely to the stressor.

In our recent paper, we studied the adaptation to repeated restraint in Fischer 344 rats with hyperreactive hypothalamic-pituitary-adrenal axis using a comparison with hyporeactive Lewis rats [11]. Fischer 344 rats subjected to a single restraint exhibited exaggerated rise in plasma ACTH, corticosterone, and adrenaline as well as a more pronounced response of blood pressure compared to Lewis rats. The repeatedly restrained Fischer 344 rats showed impaired neuroendocrine adaptation, which was accompanied by augmented stress-induced tachycardia and blunted baroreflex. From the behavioral point of view, the repeated restraint stress affected the anxiety/emotional behavior in both F344 and Lewis rats [11]. On the basis of these results, we expected impaired neuroendocrine and cardiovascular adaptation to repeated restraint stress also in SHR, but this was not the case.

In a recent review comparing multiple inbred rat strains (Brown-Norway, Fischer 344, Lewis, WKY, and SHR), Armario et al. [75] disclosed no clear relationship between endocrine response to stress and behavioral characteristics such as anxiety-like and depression-like behavior and coping style [12, 70]. Similarly, van den Buuse and Wegener [10], who examined the role of 5-HT_1A_ receptor in the cardiovascular response to stress, concluded that behavioral changes cannot explain the cardiovascular differences observed in four inbred rat strains used in their study. The same might be true for the inclusion of other control strains such as outbred Wistar or Sprague-Dawley rats.

In conclusion, stress-naive SHR exhibited several hallmarks of chronic stress corresponding to their enhanced autonomic and cardiovascular responsivity to novel stressor exposure compared with WKY rats. The repeatedly restrained WKY rats show cardiovascular and autonomic adaptation to chronic homotypic stress only during post-stress recovery period. In contrast, the repeatedly restrained SHR exhibit attenuated stress-induced tachycardia, augmented cardiac parasympathetic activity, attenuated vascular sympathetic activity, and improved baroreflex sensitivity compared to single-stressed SHR. Unexpectedly, the adaptation of cardiovascular and autonomic responses to repeated restraint was more effective in SHR. These autonomic and cardiovascular differences in the adaptation to chronic homotypic stress between SHR and WKY should be kept in mind when interpreting the results of cardiovascular experiments comprising repeated measurements of blood pressure or other stressfull manipulations with animals.

## Limitations

It might be advantageous to use the present protocol also in another control rat strain (inbred Brown Norway rats or outbred Wistar rats) for the comparison with SHR in order to eliminate a possible interference of our study with behavioral abnormalities reported in WKY rats.

## Supplementary information

Supplementary Figure1

Supplementary Table1

Supplementary Caption1

## Data Availability

The data that support the findings of this study are available from the corresponding author upon reasonable request.
