# Supplementary figures and images for: More efficient adaptation of cardiovascular response to repeated restraint in spontaneously hypertensive rats: the role of autonomic nervous system
Source: Hypertens Res. 2024 Jul 1;47(9):2377–92. doi: 10.1038/s41440-024-01765-w (PMC11374672; doi:10.1038/s41440-024-01765-w)

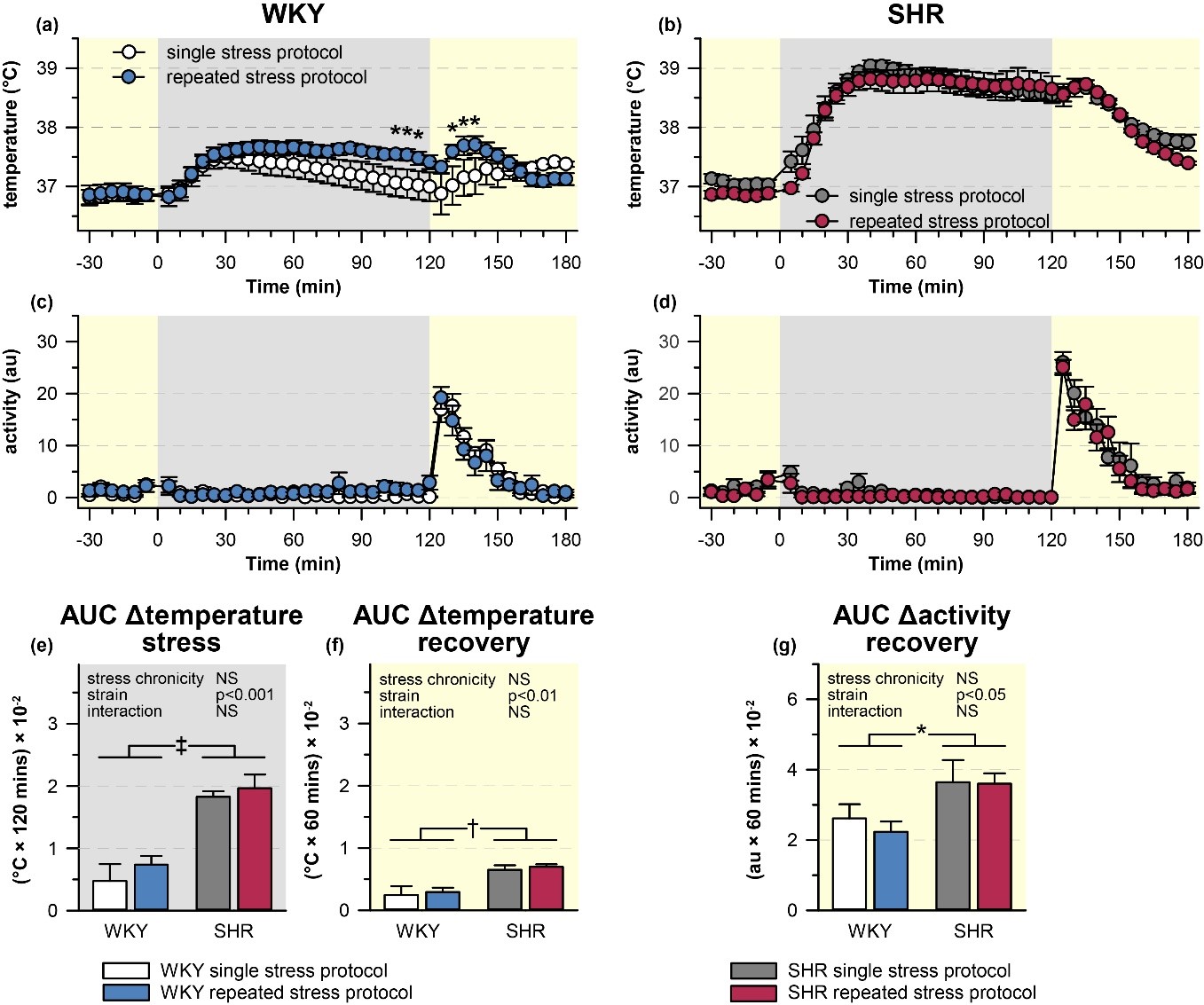

Supplement: Supplementary file 1 — Supplementary Figure1 [file 41440_2024_1765_MOESM1_ESM.jpg]
