# Supplementary material for: More efficient adaptation of cardiovascular response to repeated restraint in spontaneously hypertensive rats: the role of autonomic nervous system
Source: Hypertens Res. 2024 Jul 1;47(9):2377–92. doi: 10.1038/s41440-024-01765-w (PMC11374672; doi:10.1038/s41440-024-01765-w)
Supplement: Supplementary file 2 — Supplementary Table1 [file 41440_2024_1765_MOESM2_ESM.docx]

**Supplementary Table 1. Time-domain and frequency-domain indices of heart rate variability (HRV) during light (inactive) and dark (active) phases of the day in stress naive and chronically stressed WKY and SHR.**

|  | **WKY** | | **SHR** | | **Repeated measures**  **two-way ANOVA** | | |
| --- | --- | --- | --- | --- | --- | --- | --- |
|  | **Stress naive**  **(8)** | **Chronically stressed (8)** | **Stress naive**  **(8)** | **Chronically stressed (8)** | **Stress chronicity** | **Strain** | **Interaction** |
| **HRV time-domain indices** | | | | | | | |
| **Light (inactive) phase** | | | | | | | |
| SDNN  (ms) | 9.9 ± 0.7 | 10.2 ± 0.8 | 9.3 ± 0.6 | 9.6 ± 0.7 | NS | NS | NS |
| RMSSD  (ms) | 4.5 ± 0.4 | 4.6 ± 0.3 | 4.5 ± 0.3 | 4.8 ± 0.3 | p=0.09 | NS | NS |
| **Dark (active) phase** | | | | | | | |
| SDNN  (ms) | 8.8 ± 0.7 | 10.4 ± 0.5 | 8.9 ± 0.3 | 9.8 ± 0.6 | p<0.001 | NS | NS |
| RMSSD  (ms) | 3.8 ± 0.4 | 3.9 ± 0.2 | 3.3 ± 0.1 | 3.9 ± 0.2 ^b^ | p<0.05 | NS | p<0.05 |
| **HRV frequency-domain indices** | | | | | | | |
| **Light (inactive) phase** | | | | | | | |
| Total HRV (ms^2^) | 8.9 ± 1.6 | 10.1 ± 1.3 | 8.0 ± 0.9 | 9.2 ± 1.1 | p<0.05 | NS | NS |
| VLF-HRV (ms^2^) | 5.5 ± 1.0 | 6.3 ± 0.9 | 4.8 ± 0.6 | 5.2 ± 0.6 | NS | NS | NS |
| LF-HRV (ms^2^) | 1.1 ± 0.3 | 1.3 ± 0.2 | 0.7 ± 0.1 | 0.9 ± 0.2 | p<0.01 | NS | NS |
| HF-HRV (ms^2^) | 2.3 ± 0.4 | 2.5 ± 0.3 | 2.5 ± 0.3 | 3.1 ± 0.4 | p<0.01 | NS | NS |
| LF-HRV % (nu) | 27.2 ± 2.8 | 30.0 ± 3.1 | 21.8 ± 2.0 | 22.7 ± 1.7 | p<0.05 | p=0.08 | NS |
| LF/HF  Ratio | 0.45 ± 0.06 | 0.51 ± 0.07 | 0.32 ± 0.04 | 0.33 ± 0.04 | p=0.08 | p<0.05 | NS |
| **Dark (active) phase** | | | | | | | |
| Total HRV (ms^2^) | 8.2 ± 1.5 | 10.2 ± 1.5 | 5.8 ± 0.6 | 7.8 ± 0.9 | p<0.01 | NS | NS |
| VLF-HRV (ms^2^) | 4.9 ± 1.0 | 6.7 ± 1.1 | 3.7 ± 0.4 | 5.0 ± 0.7 | p<0.01 | NS | NS |
| LF-HRV (ms^2^) | 1.1 ± 0.2 | 1.3 ± 0.3 | 0.6 ± 0.1 | 0.8 ± 0.1 | p<0.05 | NS | NS |
| HF-HRV (ms^2^) | 2.2. ± 0.4 | 2.2 ± 0.3 | 1.5 ± 0.1^a^ | 2.1 ±0.2^b^ | p<0.05 | NS | p<0.05 |
| LF-HRV % (nu) | 30.5 ± 2.6 | 34.7 ± 3.0^b^ | 28.4 ± 2.3 | 27.7 ± 2.4 | NS | NS | p<0.05 |
| LF/HF  ratio | 0.50 ± 0.06 | 0.60 ±0.08 | 0.44 ± 0.05 | 0.44 ±0.06 | p=0.08 | NS | p=0.06 |

All parameters were determined in stress-naive and in repeatedly stressed (the first dark and light phase after daily 120 min restraint for seven consecutive days) Wistar-Kyoto (WKY) and spontaneously hypertensive rats (SHR). Average values of the parameter in particular phase are shown as the means ± SEM. Statistical significance was computed by repeated measures two-way ANOVA (factor Strain and within-subject repeated factor Stress chronicity; * P<0.05; † P<0.01; ‡ P<0.001; NS – non-significant) followed by Bonferroni *post-hoc* test. In case of significant interaction, differences between experimental groups are indicated (^a^ vs WKY rats in the same stress protocol,

^b^ vs stress-naive group of the same strain)
