# Supplementary material for: More efficient adaptation of cardiovascular response to repeated restraint in spontaneously hypertensive rats: the role of autonomic nervous system
Source: Hypertens Res. 2024 Jul 1;47(9):2377–92. doi: 10.1038/s41440-024-01765-w (PMC11374672; doi:10.1038/s41440-024-01765-w)
Supplement: Supplementary file 3 — Supplementary Caption1 [file 41440_2024_1765_MOESM3_ESM.docx]

**Supplementary Figure 1. Core body temperature and locomotor activity during restraint.** WKY and SHR were subjected to 120 min restraint (marked in grey) either once (single stress protocol) or for seven consecutive days (repeated stress protocol). The time course of changes in body temperature (a,b) and locomotor activity (c,d) were measured by radiotelemetry and averaged over five-minute intervals; each point represents a group mean ± SEM; n = 8 for each group. Area under the curve (AUC) of Δtemperature (e, f) and Δactivity (g) during the stress session (e) and post-stress recovery period (f, g). * P<0.05; † P<0.01; ‡ P<0.001 vs particular group; NS – non-significant.
